# 3,3-Dimethyl-*cis*-2,6-di-*p*-tolyl­piperidin-4-one

**DOI:** 10.1107/S160053680903579X

**Published:** 2009-09-12

**Authors:** P. Gayathri, S. S. Ilango, S. Ponnuswamy, A. Thiruvalluvar, R. J. Butcher

**Affiliations:** a PG Research Department of Physics, Rajah Serfoji Government College (Autonomous), Thanjavur 613 005, Tamilnadu, India; b Department of Chemistry, Government Arts College (Autonomous), Coimbatore 641 018, Tamilnadu, India; c Department of Chemistry, Howard University, 525 College Street NW, Washington, DC 20059, USA

## Abstract

In the title mol­ecule, C_21_H_25_NO, the piperidine ring adopts a chair conformation. The benzene rings and one of the methyl groups attached to the piperidine ring have equatorial orientations. The dihedral angle between the two benzene rings is 72.53 (9)°. In the crystal, mol­ecules are linked by N—H⋯O hydrogen bonds. Weak C—H⋯π inter­actions involving the benzene rings are also present in the crystal structure.

## Related literature

For related crystal structures, see: Gayathri *et al.* (2008 ▶); Ilango *et al.* (2008 ▶). For biological activities of piperidones, see: Aridoss *et al.* (2008 ▶). For the synthesis, see: Noller and Baliah (1948 ▶). For the stereochemistry and ring conformation of piperidin-4-ones and their derivatives, see: Ponnuswamy *et al.* (2002 ▶).
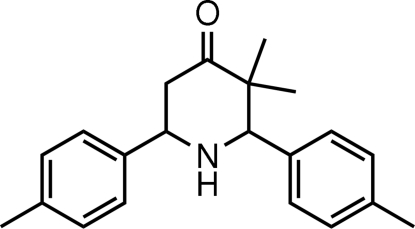


## Experimental

### 

#### Crystal data

C_21_H_25_NO
*M* *_r_* = 307.42
Orthorhombic, 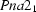

*a* = 12.9576 (3) Å
*b* = 22.6153 (5) Å
*c* = 5.9600 (1) Å
*V* = 1746.52 (6) Å^3^
*Z* = 4
Cu *K*α radiation
μ = 0.55 mm^−1^
*T* = 110 K
0.51 × 0.34 × 0.12 mm

#### Data collection

Oxford Diffraction Xcalibur diffractometer with a Ruby Gemini detector
Absorption correction: multi-scan (*CrysAlis Pro*; Oxford Diffraction, 2009 ▶) *T*_min_ = 0.665, *T*_max_ = 1.000
4198 measured reflections
1914 independent reflections
1859 reflections with *I* > 2σ(*I*)
*R*_int_ = 0.018

#### Refinement

*R*[*F* ^2^ > 2σ(*F* ^2^)] = 0.039
*wR*(*F* ^2^) = 0.106
*S* = 1.04
1914 reflections
216 parameters
1 restraint
H atoms treated by a mixture of independent and constrained refinement
Δρ_max_ = 0.26 e Å^−3^
Δρ_min_ = −0.24 e Å^−3^

### 

Data collection: *CrysAlis Pro* (Oxford Diffraction, 2009 ▶); cell refinement: *CrysAlis Pro*; data reduction: *CrysAlis Pro*; program(s) used to solve structure: *SHELXS97* (Sheldrick, 2008 ▶); program(s) used to refine structure: *SHELXL97* (Sheldrick, 2008 ▶); molecular graphics: *ORTEP-3* (Farrugia, 1997 ▶); software used to prepare material for publication: *PLATON* (Spek, 2009 ▶).

## Supplementary Material

Crystal structure: contains datablocks global, I. DOI: 10.1107/S160053680903579X/sj2644sup1.cif

Structure factors: contains datablocks I. DOI: 10.1107/S160053680903579X/sj2644Isup2.hkl

Additional supplementary materials:  crystallographic information; 3D view; checkCIF report

## Figures and Tables

**Table 1:** Hydrogen-bond geometry (Å, °)

*D*—H⋯*A*	*D*—H	H⋯*A*	*D*⋯*A*	*D*—H⋯*A*
N1—H1⋯O4^i^	0.85 (3)	2.26 (3)	3.057 (2)	157 (2)
C16—H16*B*⋯*Cg*1^ii^	0.98	2.80	3.704 (2)	154
C32—H32*A*⋯*Cg*2^iii^	0.98	2.90	3.659 (2)	135

Symmetry codes: (i) 
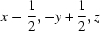
; (ii) 
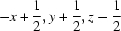
; (iii) 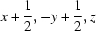. *Cg*1 and *Cg*2 are the centroids of the C21—C26 and C61—C66 rings, respectively.

**Fig. 1.**
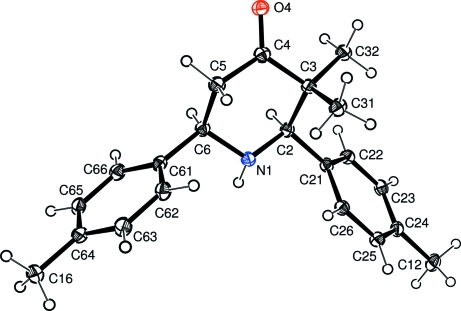
The molecular structure of the title compound, showing the atom-numbering scheme and displacement ellipsoids drawn at the 30% probability level. H atoms are shown as small spheres of arbitrary radius.

**Fig. 2.**
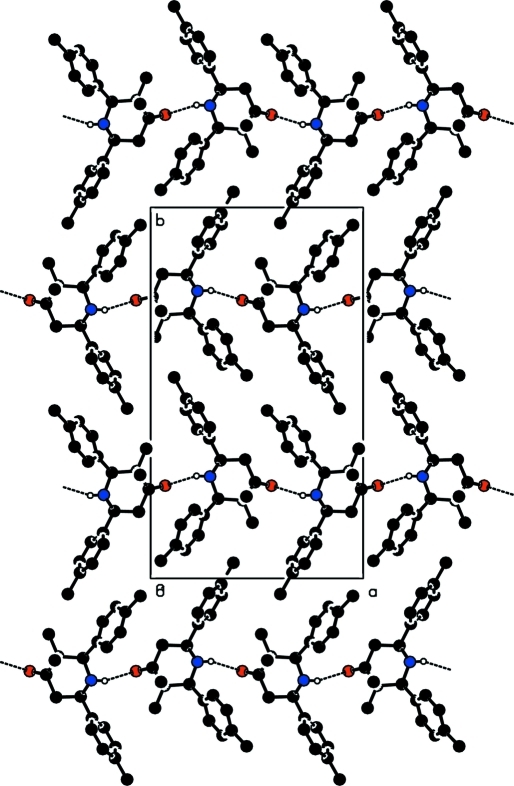
The molecular packing of the title compound, viewed down the c axis. Dashed lines indicate hydrogen bonds. H atoms not involved in hydrogen bonding have been omitted.

## supplementary crystallographic information

### Comment

Piperidin-4-ones and their derivatives show a broad spectrum of biological activity which includes antimicrobial, antiviral, anti tuberculosis and anticancer activities (Aridoss *et al.*, 2008). Recent research effort has been devoted to the discovery of potential 2,6-diarylpiperidin-4-one based chemical entities and establishing their stereochemistry, (Ponnuswamy *et al.*, 2002) because, the pharmacological effects of potential drugs depends sensitively on the stereochemistry and ring conformations.

Crystal structures of r-2,c-6-Bis(4-chlorophenyl)-t-3-isopropyl-1-nitrosopiperidin-4-one (Gayathri *et al.*, 2008) and r-2,c-6-Bis(4-chlorophenyl)-c-3,t-3- dimethylpiperidin-4-one(Ilango *et al.*, 2008) have been reported, wherein the piperidine rings adopt chair conformations.

In the title molecule, C_21_H_25_NO, Fig.1, the piperidine ring adopts a chair conformation. The benzene rings at position 2,6 and one of the methyl groups attached to the piperidine ring in 3, have equatorial orientations. The dihedral angle between the two benzene rings is 72.53 (9)°. Molecules are linked by intermolecular N1—H1···O4 (-1/2 + *x*, 1/2 - *y*, *z*)hydrogen bonds, forming an infinite one-dimensional chain with base vector [1 0 0]. Further, C16—H16B···π (1/2 - *x*, 1/2 + *y*, -1/2 + *z*) and C32—H32A···π (1/2 + *x*, 1/2 - *y*, *z*) interactions involving the benzene rings at position 2 (C21—C26) and 6 (C61—C66) are also present in the crystal structure.

### Experimental

The procedure adopted by Noller & Baliah (1948) was followed for the preparation of the title compound. To the solution of ammonium acetate (3.85 g, 0.05 mol) in dry ethanol, *p*-tolualdehyde (12.0 g, 0.1 mol) and isopropyl methyl ketone (5.35 ml, 0.05 mol) were added and allowed to reflux on a water bath for 1 h. The resulting solution was kept at room temperature for overnight and the precipitated solid was filtered. The solid was crystallized using benzene - petroleum ether mixture. The yield of the product obtained was 7.36 g (48%).

### Refinement

In the absence of any anamalous scatterers in the molecule, the Friedel pairs were merged. The absolute structure in the present model has been chosen arbitrarily. Atom H1 on N1 was located in a difference Fourier map and refined isotropically. Remaining H atoms were positioned geometrically and allowed to ride on their parent atoms, with C—H = 0.95, 0.98, 0.99 and 1.00 Å for C*sp*^2^, methyl, methylene and methine C, respectively; *U*_iso_(H) = k*U*_eq_(C), where k = 1.5 for methyl and 1.2 for all other H atoms.

### Crystal data

**Table:**

C_21_H_25_NO	*D*_x_ = 1.169 Mg m^−^^3^
*M**_r_* = 307.42	Melting point: 389(1) K
Orthorhombic, *P**n**a*2_1_	Cu *K*α radiation, λ = 1.54184 Å
Hall symbol: P 2c -2n	Cell parameters from 3875 reflections
*a* = 12.9576 (3) Å	θ = 5.2–74.0°
*b* = 22.6153 (5) Å	µ = 0.55 mm^−^^1^
*c* = 5.9600 (1) Å	*T* = 110 K
*V* = 1746.52 (6) Å^3^	Rectangular-plate, colourless
*Z* = 4	0.51 × 0.34 × 0.12 mm
*F*(000) = 664	

### Data collection

**Table:**

Oxford Diffraction Xcalibur diffractometer with a Ruby Gemini detector	1914 independent reflections
Radiation source: fine-focus sealed tube	1859 reflections with *I* > 2σ(*I*)
graphite	*R*_int_ = 0.018
Detector resolution: 10.5081 pixels mm^-1^	θ_max_ = 74.1°, θ_min_ = 5.2°
ω scans	*h* = −16→8
Absorption correction: multi-scan (*CrysAlis PRO*; Oxford Diffraction, 2009)	*k* = −27→16
*T*_min_ = 0.665, *T*_max_ = 1.000	*l* = −6→7
4198 measured reflections	

### Refinement

**Table:**

Refinement on *F*^2^	Primary atom site location: structure-invariant direct methods
Least-squares matrix: full	Secondary atom site location: difference Fourier map
*R*[*F*^2^ > 2σ(*F*^2^)] = 0.039	Hydrogen site location: inferred from neighbouring sites
*wR*(*F*^2^) = 0.106	H atoms treated by a mixture of independent and constrained refinement
*S* = 1.04	*w* = 1/[σ^2^(*F*_o_^2^) + (0.0807*P*)^2^ + 0.2385*P*] where *P* = (*F*_o_^2^ + 2*F*_c_^2^)/3
1914 reflections	(Δ/σ)_max_ = 0.001
216 parameters	Δρ_max_ = 0.26 e Å^−^^3^
1 restraint	Δρ_min_ = −0.24 e Å^−^^3^

### Special details

**Table:**

Geometry. Bond distances, angles *etc*. have been calculated using the rounded fractional coordinates. All su's are estimated from the variances of the (full) variance-covariance matrix. The cell e.s.d.'s are taken into account in the estimation of distances, angles and torsion angles
Refinement. Refinement of *F*^2^ against ALL reflections. The weighted *R*-factor *wR* and goodness of fit *S* are based on *F*^2^, conventional *R*-factors *R* are based on *F*, with *F* set to zero for negative *F*^2^. The threshold expression of *F*^2^ > 2σ(*F*^2^) is used only for calculating *R*-factors(gt) *etc*. and is not relevant to the choice of reflections for refinement. *R*-factors based on *F*^2^ are statistically about twice as large as those based on *F*, and *R*- factors based on ALL data will be even larger.

### Fractional atomic coordinates and isotropic or equivalent isotropic displacement parameters (Å^2^)

**Table:**

	*x*	*y*	*z*	*U*_iso_*/*U*_eq_	
O4	0.56853 (10)	0.24999 (6)	−0.0992 (3)	0.0283 (4)	
N1	0.27975 (12)	0.27483 (7)	0.1089 (3)	0.0224 (4)	
C2	0.31792 (13)	0.21482 (8)	0.0700 (3)	0.0211 (5)	
C3	0.43464 (13)	0.21033 (8)	0.1375 (3)	0.0231 (5)	
C4	0.49127 (14)	0.25941 (8)	0.0097 (4)	0.0239 (5)	
C5	0.44461 (14)	0.32042 (8)	0.0236 (4)	0.0281 (5)	
C6	0.32911 (14)	0.31839 (8)	−0.0373 (3)	0.0236 (5)	
C12	0.04818 (17)	0.04740 (9)	0.5387 (4)	0.0352 (6)	
C16	0.11057 (17)	0.54217 (9)	0.0765 (4)	0.0342 (6)	
C21	0.24918 (13)	0.17115 (8)	0.1927 (3)	0.0213 (5)	
C22	0.22517 (14)	0.11643 (8)	0.0988 (4)	0.0242 (5)	
C23	0.16130 (14)	0.07669 (8)	0.2094 (4)	0.0259 (5)	
C24	0.11876 (14)	0.09021 (8)	0.4177 (4)	0.0259 (5)	
C25	0.14229 (15)	0.14503 (8)	0.5111 (4)	0.0259 (5)	
C26	0.20649 (14)	0.18499 (8)	0.4018 (4)	0.0230 (5)	
C31	0.45077 (15)	0.22090 (9)	0.3902 (4)	0.0286 (5)	
C32	0.47915 (15)	0.15041 (8)	0.0708 (4)	0.0295 (6)	
C61	0.27612 (14)	0.37750 (8)	−0.0106 (3)	0.0234 (5)	
C62	0.27803 (15)	0.40730 (9)	0.1934 (4)	0.0281 (5)	
C63	0.22477 (17)	0.46020 (9)	0.2216 (4)	0.0301 (6)	
C64	0.16765 (15)	0.48444 (8)	0.0463 (4)	0.0278 (5)	
C65	0.16612 (16)	0.45467 (9)	−0.1565 (4)	0.0285 (5)	
C66	0.21927 (15)	0.40169 (9)	−0.1854 (4)	0.0275 (5)	
H1	0.216 (2)	0.2763 (10)	0.077 (5)	0.023 (6)*	
H2	0.31246	0.20644	−0.09429	0.0253*	
H5A	0.48115	0.34728	−0.08076	0.0338*	
H5B	0.45294	0.33610	0.17764	0.0338*	
H6	0.32193	0.30512	−0.19655	0.0283*	
H12A	−0.02240	0.05189	0.48213	0.0528*	
H12B	0.04947	0.05577	0.70001	0.0528*	
H12C	0.07180	0.00682	0.51226	0.0528*	
H16A	0.05083	0.54316	−0.02433	0.0513*	
H16B	0.15679	0.57518	0.04106	0.0513*	
H16C	0.08706	0.54561	0.23220	0.0513*	
H22	0.25301	0.10614	−0.04335	0.0290*	
H23	0.14636	0.03963	0.14173	0.0311*	
H25	0.11384	0.15536	0.65267	0.0311*	
H26	0.22149	0.22201	0.46970	0.0276*	
H31A	0.52472	0.22461	0.42168	0.0428*	
H31B	0.42247	0.18747	0.47486	0.0428*	
H31C	0.41538	0.25733	0.43496	0.0428*	
H32A	0.55189	0.14846	0.11519	0.0441*	
H32B	0.47353	0.14536	−0.09201	0.0441*	
H32C	0.44060	0.11891	0.14654	0.0441*	
H62	0.31622	0.39132	0.31523	0.0337*	
H63	0.22739	0.48002	0.36202	0.0361*	
H65	0.12808	0.47069	−0.27848	0.0342*	
H66	0.21659	0.38193	−0.32591	0.0330*	

### Atomic displacement parameters (Å^2^)

**Table:**

	*U*^11^	*U*^22^	*U*^33^	*U*^12^	*U*^13^	*U*^23^
O4	0.0233 (6)	0.0338 (7)	0.0279 (7)	0.0004 (5)	0.0018 (6)	0.0032 (6)
N1	0.0197 (7)	0.0216 (8)	0.0258 (8)	−0.0004 (5)	0.0003 (7)	0.0018 (7)
C2	0.0223 (8)	0.0218 (8)	0.0192 (9)	−0.0003 (6)	−0.0006 (7)	−0.0001 (7)
C3	0.0201 (8)	0.0253 (9)	0.0238 (10)	0.0003 (7)	−0.0005 (7)	0.0015 (8)
C4	0.0215 (8)	0.0270 (8)	0.0233 (9)	−0.0013 (7)	−0.0022 (8)	−0.0006 (8)
C5	0.0240 (8)	0.0234 (9)	0.0370 (11)	−0.0022 (7)	0.0040 (8)	0.0027 (8)
C6	0.0256 (9)	0.0220 (8)	0.0231 (9)	0.0003 (7)	0.0014 (7)	0.0001 (7)
C12	0.0374 (10)	0.0292 (9)	0.0390 (12)	−0.0069 (8)	0.0044 (10)	0.0088 (10)
C16	0.0349 (10)	0.0258 (9)	0.0419 (13)	0.0039 (7)	0.0066 (10)	0.0033 (9)
C21	0.0199 (8)	0.0218 (8)	0.0222 (9)	0.0011 (6)	−0.0017 (7)	0.0022 (8)
C22	0.0247 (8)	0.0249 (9)	0.0229 (9)	0.0009 (6)	−0.0008 (8)	−0.0023 (8)
C23	0.0285 (8)	0.0196 (8)	0.0297 (10)	−0.0008 (7)	−0.0045 (8)	−0.0002 (8)
C24	0.0234 (8)	0.0242 (8)	0.0302 (10)	−0.0009 (7)	−0.0015 (8)	0.0065 (8)
C25	0.0260 (9)	0.0284 (9)	0.0232 (9)	0.0017 (7)	0.0017 (8)	0.0012 (8)
C26	0.0238 (8)	0.0207 (8)	0.0245 (9)	0.0006 (6)	−0.0002 (8)	−0.0011 (8)
C31	0.0261 (9)	0.0365 (10)	0.0232 (9)	−0.0027 (8)	−0.0045 (8)	0.0023 (9)
C32	0.0263 (9)	0.0251 (9)	0.0370 (11)	0.0023 (7)	0.0033 (8)	0.0027 (9)
C61	0.0242 (8)	0.0218 (8)	0.0242 (10)	−0.0022 (7)	0.0047 (8)	0.0032 (8)
C62	0.0330 (9)	0.0271 (9)	0.0243 (10)	0.0007 (7)	−0.0023 (9)	0.0037 (9)
C63	0.0388 (10)	0.0257 (9)	0.0257 (10)	−0.0011 (8)	0.0019 (9)	−0.0023 (8)
C64	0.0260 (8)	0.0229 (9)	0.0344 (11)	−0.0018 (7)	0.0069 (8)	0.0037 (9)
C65	0.0301 (9)	0.0284 (9)	0.0270 (10)	0.0000 (7)	0.0009 (8)	0.0085 (8)
C66	0.0315 (10)	0.0284 (9)	0.0226 (9)	−0.0016 (8)	0.0013 (8)	0.0008 (8)

### Geometric parameters (Å, °)

**Table:**

O4—C4	1.212 (2)	C65—C66	1.393 (3)
N1—C2	1.463 (2)	C2—H2	1.0000
N1—C6	1.463 (2)	C5—H5A	0.9900
N1—H1	0.85 (3)	C5—H5B	0.9900
C2—C21	1.518 (2)	C6—H6	1.0000
C2—C3	1.568 (2)	C12—H12A	0.9800
C3—C4	1.533 (3)	C12—H12B	0.9800
C3—C31	1.539 (3)	C12—H12C	0.9800
C3—C32	1.525 (3)	C16—H16A	0.9800
C4—C5	1.509 (3)	C16—H16B	0.9800
C5—C6	1.541 (3)	C16—H16C	0.9800
C6—C61	1.511 (3)	C22—H22	0.9500
C12—C24	1.515 (3)	C23—H23	0.9500
C16—C64	1.511 (3)	C25—H25	0.9500
C21—C22	1.393 (3)	C26—H26	0.9500
C21—C26	1.399 (3)	C31—H31A	0.9800
C22—C23	1.388 (3)	C31—H31B	0.9800
C23—C24	1.392 (3)	C31—H31C	0.9800
C24—C25	1.393 (3)	C32—H32A	0.9800
C25—C26	1.390 (3)	C32—H32B	0.9800
C61—C66	1.388 (3)	C32—H32C	0.9800
C61—C62	1.390 (3)	C62—H62	0.9500
C62—C63	1.391 (3)	C63—H63	0.9500
C63—C64	1.393 (3)	C65—H65	0.9500
C64—C65	1.384 (3)	C66—H66	0.9500
			
O4···C21^i^	3.419 (2)	H5B···C24^i^	3.0700
O4···N1^i^	3.057 (2)	H6···H2	2.3200
O4···H32B	2.6700	H6···H66	2.3400
O4···H32A	2.6400	H12B···H25	2.4200
O4···H1^i^	2.26 (3)	H12C···H23	2.5200
O4···H25^ii^	2.6700	H16A···H65	2.4500
N1···O4^iii^	3.057 (2)	H16B···C23^vii^	3.0800
N1···H31C	2.6500	H16B···C24^vii^	3.0200
N1···H62	2.9500	H16B···C25^vii^	3.0500
N1···H26	2.5700	H16C···H63	2.4700
C21···O4^iii^	3.419 (2)	H22···H2	2.4100
C22···C32	3.384 (3)	H23···H12C	2.5200
C26···C31	3.269 (3)	H25···H12B	2.4200
C31···C26	3.269 (3)	H25···O4^viii^	2.6700
C32···C22	3.384 (3)	H25···H5A^viii^	2.3400
C4···H1^i^	3.05 (3)	H26···N1	2.5700
C5···H62	2.8900	H26···C31	3.0100
C5···H31C	2.8600	H31A···H32A	2.5400
C21···H32C	2.7600	H31B···C21	2.8300
C21···H31B	2.8300	H31B···C26	2.8300
C22···H32C	2.8100	H31B···H32C	2.5100
C23···H16B^iv^	3.0800	H31C···N1	2.6500
C24···H5B^iii^	3.0700	H31C···C5	2.8600
C24···H16B^iv^	3.0200	H31C···H5B	2.4000
C25···H16B^iv^	3.0500	H32A···O4	2.6400
C26···H1	2.83 (3)	H32A···H31A	2.5400
C26···H31B	2.8300	H32A···C61^i^	3.0600
C31···H5B	2.9000	H32A···C66^i^	3.0300
C31···H26	3.0100	H32B···O4	2.6700
C61···H32A^iii^	3.0600	H32B···H2	2.5000
C62···H5B	2.7800	H32C···C21	2.7600
C62···H66^v^	3.0300	H32C···C22	2.8100
C65···H63^vi^	3.0300	H32C···H31B	2.5100
C66···H32A^iii^	3.0300	H62···N1	2.9500
H1···C26	2.83 (3)	H62···C5	2.8900
H1···O4^iii^	2.26 (3)	H62···H5B	2.3200
H1···C4^iii^	3.05 (3)	H62···H66^v^	2.5100
H2···H6	2.3200	H63···C65^v^	3.0300
H2···H22	2.4100	H63···H16C	2.4700
H2···H32B	2.5000	H63···H65^v^	2.5100
H5A···H25^ii^	2.3400	H65···H16A	2.4500
H5B···C31	2.9000	H65···H63^vi^	2.5100
H5B···C62	2.7800	H66···C62^vi^	3.0300
H5B···H31C	2.4000	H66···H6	2.3400
H5B···H62	2.3200	H66···H62^vi^	2.5100
			
C2—N1—C6	112.50 (15)	C6—C5—H5B	110.00
C6—N1—H1	105.4 (18)	H5A—C5—H5B	108.00
C2—N1—H1	109.3 (16)	N1—C6—H6	109.00
N1—C2—C21	109.20 (14)	C5—C6—H6	109.00
N1—C2—C3	110.21 (14)	C61—C6—H6	109.00
C3—C2—C21	113.59 (14)	C24—C12—H12A	109.00
C2—C3—C31	111.83 (14)	C24—C12—H12B	109.00
C2—C3—C4	106.69 (14)	C24—C12—H12C	109.00
C4—C3—C32	109.43 (15)	H12A—C12—H12B	109.00
C31—C3—C32	109.98 (16)	H12A—C12—H12C	109.00
C4—C3—C31	107.98 (16)	H12B—C12—H12C	109.00
C2—C3—C32	110.81 (15)	C64—C16—H16A	109.00
O4—C4—C5	121.41 (18)	C64—C16—H16B	109.00
O4—C4—C3	122.29 (16)	C64—C16—H16C	109.00
C3—C4—C5	116.30 (16)	H16A—C16—H16B	109.00
C4—C5—C6	110.43 (15)	H16A—C16—H16C	109.00
N1—C6—C5	107.73 (15)	H16B—C16—H16C	109.00
C5—C6—C61	112.97 (15)	C21—C22—H22	119.00
N1—C6—C61	109.54 (15)	C23—C22—H22	119.00
C2—C21—C26	121.04 (16)	C22—C23—H23	119.00
C2—C21—C22	121.03 (17)	C24—C23—H23	119.00
C22—C21—C26	117.92 (17)	C24—C25—H25	119.00
C21—C22—C23	121.2 (2)	C26—C25—H25	119.00
C22—C23—C24	121.15 (18)	C21—C26—H26	120.00
C23—C24—C25	117.72 (18)	C25—C26—H26	120.00
C12—C24—C25	120.7 (2)	C3—C31—H31A	109.00
C12—C24—C23	121.55 (17)	C3—C31—H31B	109.00
C24—C25—C26	121.5 (2)	C3—C31—H31C	109.00
C21—C26—C25	120.57 (18)	H31A—C31—H31B	109.00
C62—C61—C66	118.34 (18)	H31A—C31—H31C	109.00
C6—C61—C62	120.85 (16)	H31B—C31—H31C	109.00
C6—C61—C66	120.71 (17)	C3—C32—H32A	109.00
C61—C62—C63	120.9 (2)	C3—C32—H32B	109.00
C62—C63—C64	120.8 (2)	C3—C32—H32C	109.00
C16—C64—C65	121.2 (2)	H32A—C32—H32B	109.00
C16—C64—C63	120.7 (2)	H32A—C32—H32C	109.00
C63—C64—C65	118.12 (18)	H32B—C32—H32C	109.00
C64—C65—C66	121.3 (2)	C61—C62—H62	120.00
C61—C66—C65	120.6 (2)	C63—C62—H62	120.00
N1—C2—H2	108.00	C62—C63—H63	120.00
C3—C2—H2	108.00	C64—C63—H63	120.00
C21—C2—H2	108.00	C64—C65—H65	119.00
C4—C5—H5A	110.00	C66—C65—H65	119.00
C4—C5—H5B	110.00	C61—C66—H66	120.00
C6—C5—H5A	110.00	C65—C66—H66	120.00
			
C6—N1—C2—C3	65.65 (19)	N1—C6—C61—C62	63.7 (2)
C6—N1—C2—C21	−168.91 (14)	N1—C6—C61—C66	−112.47 (19)
C2—N1—C6—C5	−64.42 (19)	C5—C6—C61—C62	−56.4 (2)
C2—N1—C6—C61	172.34 (14)	C5—C6—C61—C66	127.44 (19)
N1—C2—C3—C4	−53.85 (19)	C2—C21—C22—C23	−179.21 (17)
N1—C2—C3—C31	64.01 (19)	C26—C21—C22—C23	−0.3 (3)
N1—C2—C3—C32	−172.90 (16)	C2—C21—C26—C25	178.97 (17)
C21—C2—C3—C4	−176.75 (15)	C22—C21—C26—C25	0.1 (3)
C21—C2—C3—C31	−58.9 (2)	C21—C22—C23—C24	0.2 (3)
C21—C2—C3—C32	64.2 (2)	C22—C23—C24—C12	179.41 (19)
N1—C2—C21—C22	142.56 (17)	C22—C23—C24—C25	0.1 (3)
N1—C2—C21—C26	−36.3 (2)	C12—C24—C25—C26	−179.65 (19)
C3—C2—C21—C22	−94.0 (2)	C23—C24—C25—C26	−0.4 (3)
C3—C2—C21—C26	87.2 (2)	C24—C25—C26—C21	0.3 (3)
C2—C3—C4—O4	−129.7 (2)	C6—C61—C62—C63	−176.61 (18)
C2—C3—C4—C5	49.4 (2)	C66—C61—C62—C63	−0.3 (3)
C31—C3—C4—O4	109.9 (2)	C6—C61—C66—C65	176.65 (18)
C31—C3—C4—C5	−71.0 (2)	C62—C61—C66—C65	0.4 (3)
C32—C3—C4—O4	−9.8 (3)	C61—C62—C63—C64	0.4 (3)
C32—C3—C4—C5	169.34 (18)	C62—C63—C64—C16	−179.47 (19)
O4—C4—C5—C6	127.7 (2)	C62—C63—C64—C65	−0.5 (3)
C3—C4—C5—C6	−51.4 (2)	C16—C64—C65—C66	179.51 (19)
C4—C5—C6—N1	54.8 (2)	C63—C64—C65—C66	0.5 (3)
C4—C5—C6—C61	175.93 (17)	C64—C65—C66—C61	−0.5 (3)

Symmetry codes: (i) *x*+1/2, −*y*+1/2, *z*; (ii) *x*+1/2, −*y*+1/2, *z*−1; (iii) *x*−1/2, −*y*+1/2, *z*; (iv) −*x*+1/2, *y*−1/2, *z*+1/2; (v) *x*, *y*, *z*+1; (vi) *x*, *y*, *z*−1; (vii) −*x*+1/2, *y*+1/2, *z*−1/2; (viii) *x*−1/2, −*y*+1/2, *z*+1.

### Hydrogen-bond geometry (Å, °)

**Table:**

*D*—H···*A*	*D*—H	H···*A*	*D*···*A*	*D*—H···*A*
N1—H1···O4^iii^	0.85 (3)	2.26 (3)	3.057 (2)	157 (2)
C16—H16B···Cg1^vii^	0.98	2.80	3.704 (2)	154
C32—H32A···Cg2^i^	0.98	2.90	3.659 (2)	135

Symmetry codes: (iii) *x*−1/2, −*y*+1/2, *z*; (vii) −*x*+1/2, *y*+1/2, *z*−1/2; (i) *x*+1/2, −*y*+1/2, *z*.
